# Understanding the variation of modern endoscopic ultrasound use in patients with oesophageal cancer (VALUE): protocol for a multi-methods study

**DOI:** 10.1093/bjro/tzaf012

**Published:** 2025-05-21

**Authors:** Kieran G Foley, Cherish Boxall, James Franklin, Andrew Cook, Tim Underwood, Gareth Griffiths, Kelly Cozens, Katherine Bradbury, Margaret Fay, David Chuter, Kerry-Ann Longman, Ben Lindfield, Chris Hurt

**Affiliations:** Division of Cancer & Genetics, School of Medicine, Cardiff University, Cardiff, CF14 4XN, United Kingdom; Cancer Research UK Southampton Clinical Trials Unit, University of Southampton and University Hospital Southampton NHS Foundation Trust, SO16 6YD, United Kingdom; Institute of Medical Imaging and Visualisation, Bournemouth University, Bournemouth, BH12 5BB, United Kingdom; Cancer Research UK Southampton Clinical Trials Unit, University of Southampton and University Hospital Southampton NHS Foundation Trust, SO16 6YD, United Kingdom; School of Cancer Sciences, Faculty of Medicine, University of Southampton, Southampton, SO17 1BJ, United Kingdom; Cancer Research UK Southampton Clinical Trials Unit, University of Southampton and University Hospital Southampton NHS Foundation Trust, SO16 6YD, United Kingdom; Cancer Research UK Southampton Clinical Trials Unit, University of Southampton and University Hospital Southampton NHS Foundation Trust, SO16 6YD, United Kingdom; Cancer Research UK Southampton Clinical Trials Unit, University of Southampton and University Hospital Southampton NHS Foundation Trust, SO16 6YD, United Kingdom; Patient Representative, United Kingdom; Patient Representative, United Kingdom; Cancer Research UK Southampton Clinical Trials Unit, University of Southampton and University Hospital Southampton NHS Foundation Trust, SO16 6YD, United Kingdom; Cancer Research UK Southampton Clinical Trials Unit, University of Southampton and University Hospital Southampton NHS Foundation Trust, SO16 6YD, United Kingdom; Cancer Research UK Southampton Clinical Trials Unit, University of Southampton and University Hospital Southampton NHS Foundation Trust, SO16 6YD, United Kingdom

**Keywords:** oesophageal neoplasm, endoscopic ultrasound, diagnosis, staging, metastases, imaging, management, qualitative

## Abstract

**Objectives:**

Over 9000 patients are diagnosed with oesophageal cancer annually in the United Kingdom (UK). Decision-making about treatment options is influenced by radiological staging, which may include computed tomography (CT), positron emission tomography (PET), and endoscopic ultrasound (EUS). The use of EUS varies considerably around the UK and, since the introduction of PET-CT, the added value of EUS has been questioned. The VALUE study aims to understand this variation and determine how often and why EUS changes treatment decisions. VALUE will also evaluate patient and clinician experiences and opinions of EUS.

**Methods:**

This is a prospective, observational study investigating EUS in oesophageal cancer staging. Patients will be recruited at up to eleven sites in the UK, where they will be consented (if eligible) and registered onto iMedidata RAVE. Clinical and demographic data, TNM staging, pre and post EUS treatment decisions, and complications will be collected. We will attempt to sample patients from ethnic minority backgrounds in the study population, as they are underrepresented in research. Up to 30 patients and 30 clinicians will be interviewed to evaluate the use of EUS and experiences of both patient and clinician. The primary endpoint is the proportion of cases that EUS changes treatment decisions. Secondary endpoints include identification of factors that clinicians’ and patients consider when deciding if EUS should be used, the time from diagnosis to treatment decision before and after EUS, and the reasons why EUS changed management. The study has been registered on Clinicaltrials.gov: NCT06440174. The trial is open to recruitment.

**Results:**

In total, 180 patients with potentially curable oesophageal cancer who are suitable for EUS will participate. Recruitment is currently planned until September 2025 and study results will be reported after June 2026.

**Conclusion:**

The VALUE study will enable a better understanding of how and why EUS is used in oesophageal cancer. This research will identify important factors that clinicians and patients consider when deciding EUS use and determine the frequency that EUS changes treatment decisions in the modern staging pathway.

**Advances in knowledge:**

The VALUE study is a prospective, multi-centre observational study investigating the use of EUS in the modern era of oesophageal cancer staging. The study aims to determine how often and why EUS changes treatment decisions. A qualitative component will explore both clinician and patient attitudes towards EUS.

## Introduction

Over 9000 patients are diagnosed with oesophageal cancer in the United Kingdom (UK) annually. The prognosis of these patients is poor, with an overall 5-year survival rate of 15%.^1^ Most patients (60%) present with advanced disease and palliation is the only treatment option. Accordingly, oesophageal cancer has considerable unmet research need.^2^

Shared decision-making about treatment options in oesophageal cancer is largely influenced by radiological staging, which inform clinicians of the likely disease extent,^3^ in combination with histopathology, and patient factors. Radiological staging may include computed tomography (CT), positron emission tomography (PET), and endoscopic ultrasound (EUS) which provide complementary information, yet each are affected by technical limitations. These tests help determine whether radical treatment is attempted, using either curative surgery or definitive chemoradiotherapy, or if palliation is most appropriate.

CT is a standard staging investigation for all patients with oesophageal cancer, and PET-CT is recommended in patients with potentially curable disease,^4^ except in suspected T1 tumours.^5^ In contrast, national guidance for EUS staging is less clear, and as a result, there is considerable variation in EUS utilisation. A survey of oesophageal cancer multi-disciplinary team (MDT) leaders across the UK,^6^ including 35 responses representing 97 UK NHS trusts, found that 29% recommended EUS for all potentially curable patients, whereas 46% requested EUS after PET-CT on a case-by-case basis. 20% reported both a lack of utility and concerns about treatment delay. 63% and 43% routinely use EUS for radiotherapy and surgical planning, respectively. Data from the National Oesophago-Gastric Cancer Audit (NOGCA) demonstrate a decline in EUS use from 62% of all patients in 2013, to 39% in 2019, and 18.6% to 2021,^7^ although there may have been an impact of the COVID-19 pandemic on the 2021 data. In 2020/21, EUS was used in 23.6% of patients who had a curative treatment plan.

Whilst the use of EUS is declining, the use of PET-CT for oesophageal cancer staging is increasing.^7^ However, evidence for this change in practice is limited. It is unclear whether reduced EUS utilisation is due to capacity constraints, and is therefore being used pragmatically, rather than in all patients who may benefit.^8^

National variation in staging pathways is undesirable. Patients should not undergo tests from which they do not benefit nor have treatment unnecessarily delayed. Equally, inconsistent radiological staging could cause variable patient selection for radical treatment, or variable delivery of treatment. There are also cost implications for healthcare services which should make responsible use of its resources. Therefore, the issue of unequal access to diagnostic tests must be addressed.

VALUE is a prospective observational study investigating EUS in the modern era of oesophageal cancer staging. A quantitative study component will examine how often and why EUS changes treatment decisions after initial staging with CT and PET-CT. A qualitative study component will explore both clinician and patient attitudes and opinions towards the utility of EUS in the staging pathway.

## Methods

### Study design

**Figure 1.**
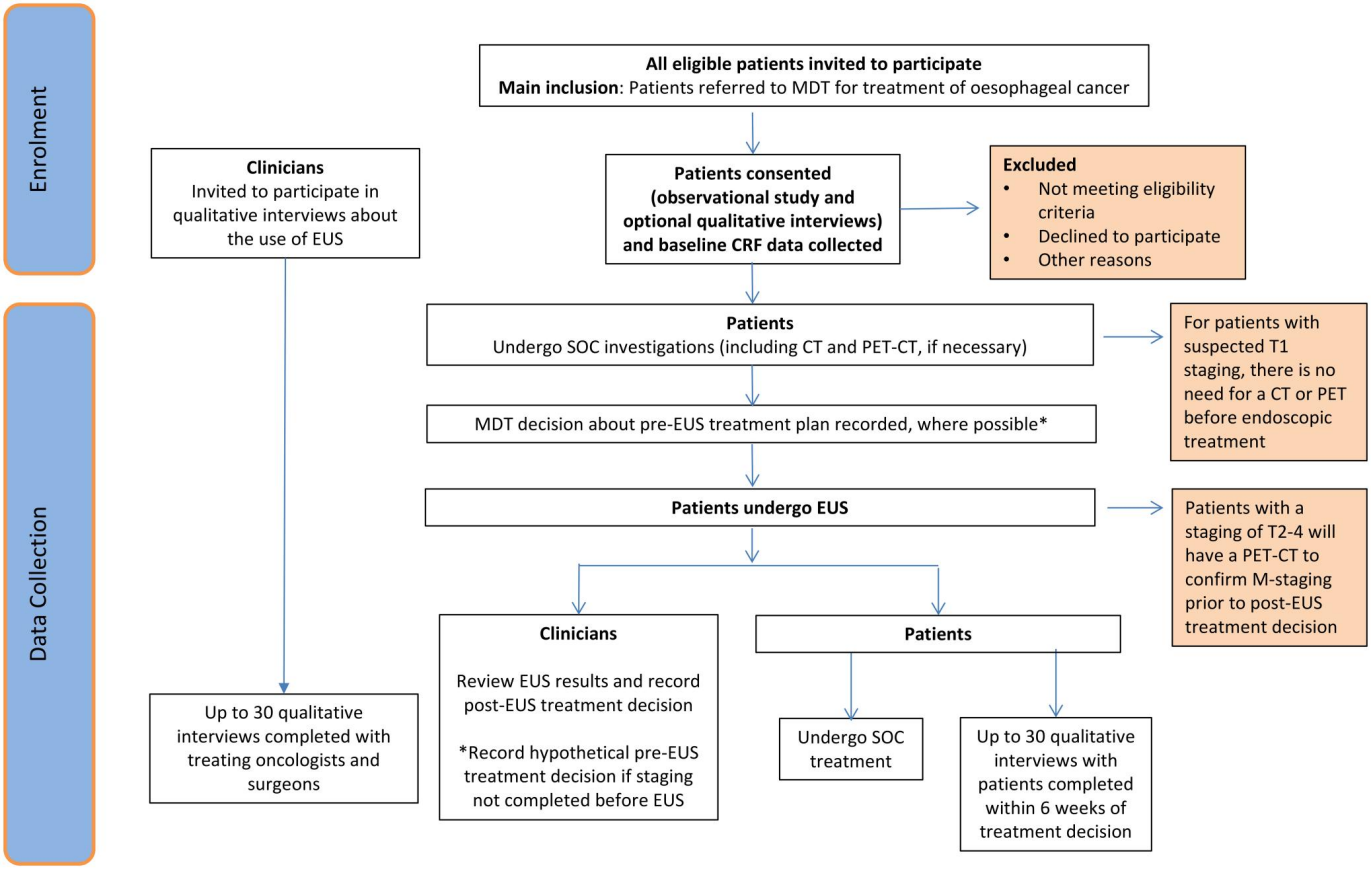
Trial schema.

Co-ordinated by the Cancer Research UK Southampton Clinical Trials Unit, this is a prospective, multi-centre, observational cohort study using a mixed-methods design (Figure 1). Both quantitative and qualitive components are planned within VALUE (REC: 24/WS/0021). In total, 180 patients with biopsy-confirmed oesophageal or junctional cancer who are deemed to have potentially curable disease, and who are intended to receive EUS as part of their standard of care staging pathway, will be recruited from clinical centres in the UK. Up to 30 clinicians who regularly care for oesophageal cancer patients in a multi-disciplinary setting and up to 30 patients will be interviewed to gather their opinions and experiences of EUS. In the context of PET-CT, EUS is considered stage 2b following the model suggested by the IDEAL framework.^9^ The study is being sponsored by University Hospital Southampton and ethically approved, by the West of Scotland Research Ethics Committee. The NIHR has funded the study (NIHR reference number: 204931).

### Participant screening

Patients will be recruited through MDTs at participating secondary care centres in the UK. Those deemed suitable for the study will be approached by their direct care team to participate. Eligibility criteria are listed in Table 1. Patients will be screened for eligibility, consented, and registered on the trial specific electronic Case Report Form (eCRF), where EUS is recommended. All patients will have the following data collected at screening to confirm eligibility; informed consent (can be received remotely), eligibility evaluation, medical history, Eastern Cooperative Oncology Group (ECOG) performance status 0-2, and clinical staging of disease by CT scan (if applicable). Patients may undergo PET-CT before EUS referral or before consent. Patients who have definite M1 disease on PET-CT, or a total length of disease deemed unsuitable for planned radical treatment by the MDT, are not eligible. The patient will be asked for consent to take part in the research before the EUS procedure (supplementary material). We ask that patients have at least 24 h to read the patient information sheet before giving consent, but can give consent on the day of the EUS, provided they have read the patient information sheet at least 24 h prior. To take part in the qualitative interviews, patients will sign an optional part of the consent form which will give the qualitative researcher permission to invite them to interview.

**Table 1.** Eligibility criteria.

Inclusion criteria	Exclusion criteria
Patients aged 16 or above with first diagnosis of biopsy-confirmed oesophageal cancer Referred for EUS examination as part of standard of care investigations Tumour location in the oesophagus, or gastro-oesophageal junction (Siewert types I-III) MDT decision that patient is potentially curable with radical treatment (e.g., endoscopic treatment, surgery +/− neoadjuvant therapy, or definitive chemoradiotherapy) Eastern Cooperative Oncology Group (ECOG) performance status 0-2 Either: Clinical staging of T1 disease (CT and PET-CT are not required) Or: Clinical staging of T2-T4, N0-N3, M0 disease confirmed by CT scan. Adenocarcinoma or squamous cell carcinoma (SCC) histopathological cell type	Recurrent or residual disease Known distant metastatic disease Previous oesophagectomy or oesophageal radiotherapy Unable to undergo EUS examination Other histopathological cell type

### Quantitative study

#### Primary objective

- To determine the proportion of cases in which EUS changes disease management.

#### Secondary objectives

- To determine how and why EUS changed the management.
- To determine time from diagnosis to treatment decision before and after EUS.

#### Data collection

The following information will be entered onto the eCRF following the EUS procedure:

- EUS report (details of what is recorded in the report should be recorded in Rave)
- Post EUS Treatment Plan agreed with patient.
- EUS complications (bleeding, infection, damage to teeth, aspiration, adverse reaction to sedation, perforation). Any complications occurring within the first 2 weeks following EUS will be recorded.
- If the treatment plan changed, details of reason(s) why EUS +/− FNA changed the treatment plan should be recorded.

Details of the treatment the patient receives will be recorded, and could include surgery, chemoradiotherapy (CRT) or chemotherapy. Data will be added within 6 months of registration.

Participation data will be entered remotely at site and retained with current data protection regulations. The principal investigator (PI) at each site is responsible for ensuring the accuracy, completeness and timelines of the data entered. Each participant is assigned a participant identifier code which is used to identify the participant during trial between the SCTU and site.

#### Primary endpoint

- Percentage of patients where treatment plan changes post-EUS.

#### Secondary endpoints

- To identify factors that clinicians and patients consider when deciding whether EUS should be used.
- How and why EUS changed the management.
- Time from diagnosis to treatment decision before and after EUS.

### Statistical analysis

#### Primary endpoint

We will compare the proportion of cases where EUS changes MDT decision against a null hypothesis of 5% with a one-sided test. We will also present 90% confidence intervals around the estimated proportion using the Wilson method.

#### Secondary endpoint

Where management was changed, we will tabulate reasons for how and why using descriptive statistics. We will calculate the time from treatment decision prior to EUS to treatment decision post EUS to measure the delay generated by waiting for an EUS. We will investigate whether patient and/or centre factors are associated with longer delay using cox regression methods with centre as a shared frailty.

#### Sample size calculation

The sample size is based on estimating the proportion of cases that EUS (when recommended) changes MDT decisions regarding treatment. We will test the observed proportion against a null hypothesis of 5% (considered too small to be of clinical use) using an alternative hypothesis of 10% (considered to be the level at which EUS may be beneficial). With 180 participants, we have 85% power based on a one-sided test with 5% type I error rate (STATA SE 18).

### Qualitative study

#### Objective

- To explore factors that influence clinicians and patients’ decision-making about whether EUS should be used.

#### Methods

In parallel to the observational study, semi-structured interviews will be conducted remotely (over the telephone or by video call) as per participant preference.

#### Patient interviews

##### Recruitment

Patient interviews will be up to 60 min with up to 30 trial participants who have optionally consented to interviews. Patients will be invited (via information sheet and informed consent form) to participate in an optional interview in conjunction with the observational study. Following the sampling criteria for the observational study, patients will be contacted by the qualitative researcher anytime between consent and 6 weeks after EUS by phone or email (patient preference) to provide an opportunity to ask questions and confirm willingness to participate before scheduling an interview. Consent will be confirmed prior to interview recording.

##### Eligibility criteria

- Capacity to consent.
- Consented to participate in the observation study.
- Patients who underwent EUS in the last 6 weeks.

##### Sampling

Purposive sampling of up to 30 patients from participating sites will be interviewed to reach information of power. Interviews will initially be with locally advanced (T2+ and/or N1+) patients. If information of power is deemed to be reached prior to 20 interviews, sampling will subsequently be directed to early-stage patients to compare similarities and differences between groups. If information of power is not reached prior to 20 interviews, sampling will focus on late-stage patients only to optimise the depth and transferability of findings. A conscious effort will be made to sample patients from ethnic minority backgrounds from the baseline demographic data collected in the eCRF as they are known to be currently under-represented in cancer research.

#### Data collection

For reporting purposes demographic data including age, sex, ethnicity, and index of multiple deprivation factors (e.g., post code and education level) will be collected at interview. Interviews will be scheduled prior to treatment initiation or early in the first neoadjuvant chemotherapy cycle (usually 4-6 weeks after EUS) and completed up to 6 weeks after EUS to mitigate the attrition of memory. This approach has been supported by patient and public representatives.

A topic guide has been developed to explore patients’ experiences and factors influencing acceptability of EUS. The interview will focus on patient’s understanding of EUS, their experience of the procedure, and perspectives on how widely they feel it should be used. Patient interviews will commence before clinician interviews so that themes can be presented and discussed with clinicians to understand if/how the information might impact their EUS related decision-making.

Data from all centres will be analysed together and published as soon as possible. A detailed statistical analysis plan will be developed prior to database lock, and all data and appropriate documentation will be stored for a minimum of 25 years after the completion of the trial.

### Clinician interviews

#### Recruitment

Clinician interviews will be up to 30 min with up to 30 clinicians who have consented to interview. The following recruitment strategies will be used to identify eligible participants.

1. Invite investigators from recruiting sites.
  An invitation email with the participant information sheet (PIS) will be sent to clinicians (identified by the CI and co-apps) eligible to participate.
2. Ask investigators to identify other eligible clinicians
  Clinicians may cascade information and pass on contact details to the qualitative interviewer or share the contact details of the interviewer.
3. Advertise through appropriate networks including other UK NHS sites, who the research team already have links and through the specialist societies and Royal Colleges.

#### Eligibility

Clinicians (e.g., surgeons, clinical oncologists) responsible for deciding whether to use EUS as part of treatment planning at the time of interview.

#### Sampling

Purposive and snowball sampling will be used to recruit up to 30 clinicians for semi-structured interviews over 12 months to reach information of power. Clinicians can send an expression of interest to the interviewer, who will respond with a PIS and opportunity to ask questions. Upon acceptance to interview, the qualitative researcher will schedule a mutually convenient date and time and verbal consent will be recorded prior to interview.

### Data collection

For reporting purposes demographic and professional data including age, sex, and job role will be collected at interview. Interviews with clinicians will focus on the organisational, patient, and experiential influencers to the use of EUS.

A topic guide has been developed to understand the oesophageal cancer staging pathway currently in place at the clinician’s institution or region, and how EUS fits into this. Additionally, the guide aims to explore the various factors influencing clinicians’ decisions regarding the use of EUS for staging, such as resource availability, clinical indications, case complexity, and patient preferences.

### Patient and clinician qualitative data analysis

Interview data will be transcribed verbatim, anonymised, and analysed using an inductive thematic approach. Analysis will take place in parallel to data collection to allow for further exploration of topics of interest in relation to the research question. A coding frame will be developed from themes derived from the data^10^ with constant comparison to identify factors that influence contrasting attitudes towards the use of EUS. NVivo qualitative data management software will facilitate management of the dataset.

Independent quantitative and qualitative analyses will be performed initially, with subsequent integration of the two methodological approaches to enrich the interpretation of findings. For clinicians that use EUS routinely or regularly, we will triangulate data from both the observational study and qualitative interviews to capture how they use the results of EUS in subsequent treatment decisions. Additionally, where possible, patients' individual understanding of the reason they received EUS from interview data will be compared with the respective information the clinicians entered on the eCRF to explore insight into the adequacy of the consent process for EUS. Iterative refinement of codes and proposed themes will occur through discussion with the research team.

### Trial discontinuation and participant withdrawal

Participants may be discontinued from the trial if the participant meets an exclusion criterion (either newly developed or not previously recognized) that precludes further trial participation. Treatment data will be collected at the time of trial discontinuation. Full details of the reason for trial discontinuation will be recorded in the End of Study electronic case report form (eCRF) and the participant’s medical record.

The participant/legal representative is free to withdraw consent from the trial at any time, without providing a reason, and without their medical care or legal rights being adversely affected. Participants may be withdrawn from the study either at their own request or at the discretion of the Investigator. The participants will be made aware that this will not affect their future care. Participants will be made aware (via the information sheet and consent form) that should they withdraw the data collected to date cannot be erased and may still be used in the final analysis. Full details of the reason for trial discontinuation should be recorded in the end of study eCRF and medical record.

### End of trial

The end of trial is defined as being when the last participants data has been collected and all data required to answer the study objective has been received and reviewed.

### Oversight committees and patient and public involvement (PPI)

The Trial Management Group (TMG) is responsible for overseeing the progress of the trial. The Chair of the TMG is the Chief Investigator of the trial and the TMG includes representatives with experience in radiology, surgery and oncology as well as being supported by two Patient and Public Involvement Contributors as well as Southampton Clinical Trials Unit (SCTU) staff who are involved in the day-to-day management of the trial. Oversight of the trial is also discussed at the Trial Steering Committee (TSC) which meets bi-annually. The SCTU undertakes several internal audits of its own systems and processes annually and has routine audits from both its Sponsor and the independent MHRA.

## Discussion

The incidence of oesophageal cancer has increased in recent decades and is expected to continue growing.^11^ Oesophageal cancer treatment planning is complex and requires multi-disciplinary input to decide upon the treatment most likely to deliver the best outcome for each patient. For instance, quality of life (QoL) is only regained 2 years after oesophagectomy^12^ and 60% of radically treated patients develop disease recurrence within 3 years.^13^ Two-year survival after oesophagectomy is only 70%.^14^ Therefore, patient selection for radical treatment is crucial, and must improve.

EUS is used variably within a multi-modality approach to radiological staging and informs local tumour (T-) and node (N-) staging which are important prognostic indicators of survival.^15^ EUS is a relatively safe procedure, although there are risks of complication such as adverse reactions to sedation and oesophageal perforation, which is potentially life-threatening, if severe. EUS is a specialist investigation requiring many years of dedicated training to perform competently.

Conflicting data concerning the clinical effectiveness of EUS in oesophageal cancer staging exist. A systematic review,^16^ updating a prior review,^17^ found that current evidence concerning the impact of EUS on the management and outcome of oesophageal cancer patients in modern staging with PET-CT was of limited quality. In total, 18 studies with 11836 patients were included. Overall, 2805 patients (23.7%) underwent EUS compared to 9031 (76.3%) without. However, only 19.7% of all patients also had PET-CT for staging. Reported change of management by EUS varied widely from 0% to 56%.

The Cancer of Oesophagus or Gastricus-New Assessment of Technology of Endosonography (COGNATE) trial^18^ randomized patients between EUS with CT, and CT alone. EUS led to improved quality-adjusted survival. However, since COGNATE, oesophageal cancer staging has been transformed by PET-CT, a cross-sectional nuclear imaging test usually performed prior to EUS.^4^ PET-CT has greater sensitivity for distant metastases than CT,^19^ and therefore identifies more patients unsuitable for radical treatment, meaning that local staging with EUS becomes less critical in these patients.^20^

In contrast, a large retrospective cohort study by Findlay et al^21^ included 953 patients, of which 798 had EUS, and 918 had PET-CT. The authors found that patient management was changed by EUS in 11% of cases, but when probability thresholds were calculated, the utility of EUS in the majority of patients (71.8% staged T2-T4a) was minimal (0.4%), concluding that the risk of EUS exceeded its benefit. These data have not been validated outside of this single-centre study but does question the value of EUS in the modern staging pathway. Should EUS continue to add clinical value, then patients who have EUS omitted from their staging pathways risk receiving sub-optimal treatment decisions from incomplete staging.^22^ Conversely, if EUS is not effective, then patients may be exposed to an unnecessary invasive test with potential complications and no clear benefit, and NHS resources could be re-distributed to other patients in need.

Patient views must also be considered, but there has been a lack of patient engagement regarding EUS in oesophageal cancer. One prospective single-centre study published in 1999 examining EUS across different tumour sites investigated patient acceptance using independent questionnaires. In patients able to remember the EUS examination (42%), 90% found it tolerable, and 83% were willing to have repeated EUS.^23^ This sparse evidence must be updated and specifically related to the modern oesophageal cancer staging pathway.

The evidence concerning EUS in oesophageal cancer is conflicting, limited, outdated, and mostly low-quality. Wide variation in practice around the UK is documented and its value should be questioned given the potential impact of the intervention on patients and the delivery of care. Not all patients receive EUS due to conflicting views concerning its modern clinical effectiveness.^6^ Existing NICE guidance^4^ recommends that EUS is only used to guide ongoing management decisions. However, this guidance can be interpreted differently. The lack of high-quality evidence hinders definitive guideline development, which drives variation in clinical practice and inequality to service access. Therefore, the evidence suggests a need to investigate the current clinical effectiveness of EUS in oesophageal cancer. In addition, there is a need to explore the factors associated with EUS use. Clinician and patient factors concerning its use must be better understood to determine its utility in the NHS and standardise practice ensuring equal access for all patients.

The VALUE study will address these research needs by creating a better understanding of how and why EUS is, and should be, used. More than ever, high-quality evidence concerning the effectiveness of cancer investigations is needed during the recovery from the COVID-19 pandemic. Delays in cancer diagnostics are well-documented, therefore optimisation of these pathways must be addressed and informed by high-quality evidence. Any investigation extending the pathway perceived to have little or no value should be considered for omission. Furthermore, health care costs are rising,^24^ particularly in patients treated with radical intent, which requires intensive and expensive health-care resources.

We anticipate that this research will identify important factors that clinicians and patients consider when deciding EUS use and determine the frequency that EUS changes treatment decisions in the modern staging pathway. The results will assist the creation of effective standardised oesophageal cancer staging which would enhance patient selection for radical and palliative treatments with better outcomes for both groups and consequent health economic benefits. If high-quality evidence suggests ongoing clinical effectiveness, patients with oesophageal cancer from across the UK should have equal access to EUS services.

## Supplementary Material

tzaf012_Supplementary_Data
