# Supplementary material for: Understanding the variation of modern endoscopic ultrasound use in patients with oesophageal cancer (VALUE): protocol for a multi-methods study
Source: BJR Open. 2025 May 21;7(1):tzaf012. doi: 10.1093/bjro/tzaf012 (PMC12145170; doi:10.1093/bjro/tzaf012)
Supplement: tzaf012_Supplementary_Data [file tzaf012_supplementary_data.zip › VALUE_Patient_Information_Sheet_v4_08Apr2024_.pdf]

<Site to print on site headed paper>

Understanding the variation of modern endoscopic ultrasound use in patients with oesophageal cancer: a multi-methods study.

Patient Information Sheet

You are being invited to take part in a research study. Before you decide, it is important for you to understand why the research is being carried out and what it will involve. Please read the following information carefully and discuss it with others if you wish. Please ask if there is anything that is unclear or if you would like more information. Thank you for taking the time to read this information sheet.

Summary of information

- The trial is being coordinated by the Southampton Clinical Trials Unit (SCTU) and the Sponsor of the trial is University Hospital Southampton. The trial has been ethically reviewed by West of Scotland Research Ethics Committee.
- The study is seeking 180 patients with oesophageal cancer from clinical centres in the UK who are being referred for endoscopic ultrasound (EUS) to record how EUS influences treatment decisions.
- Participation is entirely voluntary, and you have the freedom to decide whether to take part. You can withdraw at any time without providing a reason.
- If you choose to participate, you'll need to attend one hospital visit for the study, which can be during a routine visit, where you'll sign a consent form and provide details about your medical history, any other information collected is part of your standard care.
- Up to 30 participants who have received an EUS can take part in an optional interview about their experience, providing valuable insights for doctors. Participants will receive a £25 voucher for their time.
- The study aims to improve future treatment for oesophageal cancer patients. While direct benefits to you are not expected, your involvement may contribute to advancements in care for others.

What is the purpose of the study?

FUNDED BY
NIHR | National Institute for Health and Care Research

Patients with oesophageal cancer may undergo an endoscopic ultrasound (EUS), which involves a camera with a small ultrasound probe attached being inserted through the mouth into the oesophagus and allows a detailed assessment of nearby organs and glands. Doctors may use this test to assess the extent of disease (staging) and plan treatment. The way staging happens has changed over the last 20 years and a test, called a PET scan, is now used which has changed the way patients are selected for treatment and has called into question the usefulness of EUS.

You have been invited to take part in this study because your clinician has referred you to have an endoscopic ultrasound. As part of this study, we will record how often EUS changes treatment decisions. Doctors will be asked to record which treatment they would have recommended before the EUS. This will be compared to the actual treatment that you receive after the EUS. The treatment choice recorded before your EUS is for research only and does not mean that you will receive this treatment. These decisions will help us understand how effective EUS is.

Who can participate in the study?

We are asking 180 patients, with oesophageal cancer, from clinical centres in the UK to participate in this study.

Do I have to take part?

No, it is up to you to decide if you would like to take part. We will describe the study in detail and go through this information sheet with you. If you agree to take part, we will ask you to sign a consent form with the Doctor. You are free to withdraw at any time, without giving us a reason.

What will happen to me if I take part?

If you agree to take part, you will only need to come into hospital for your standard care assessments. Consent can be taken during one of these visits (before the EUS) or can be done via post or email. We will also ask for some details about your medical history at this visit. You will undergo an EUS as part of standard care. Taking part in the trial will not influence the decision of your care team or your treatment plan. We would like to collect the information from the scan and if/how your treatment plan may change following the scan. Following this we will collect the details of your treatment from your medical notes. You will not need to attend hospital for any additional visits above standard of care for the trial, following the initial consent visit.

What are the possible benefits of taking part?

We hope the information we get will improve treatment for patients with oesophageal cancer in the future. You may find talking through your experiences beneficial but no direct benefit to you is expected. It may however help many others in the future.

What are the possible disadvantages and risks of taking part?

There will be no greater risk than usual standard care by taking part in this trial.

Optional Interview

FUNDED BY NIHR | National Institute for Health and Care Research

We would like to discuss your experience and thoughts on the EUS procedure. The discussion is confidential and one-to-one with an independent member of the academic research team who is experienced in this type of research. The research team are not medical doctors. It will take up to one hour. It could help doctors understand how easy or hard the procedure is for patients and if you think doctors should consider this when deciding to recommend it to other patients. We'll be talking to up to 30 people, you can indicate your voluntary consent for the interview on the consent form. If you consent to be contacted the researcher will call you to discuss any queries and find a convenient time to talk to you. It may be that you are not contacted because we have reached our maximum number of interviews.

Expenses and payment

You will not receive any payment for taking part in the main trial. If you complete the optional interview, you will receive a £25 voucher for your time. The voucher can be spent in multiple locations (e.g., Supermarket, Bookstore, Clothes store) and can be posted or emailed to you.

There are no travel expenses for this study. Consent can be taken over the phone and the signed form returned via email of post to the hospital or can be done in person during one of your routine visits. Interviews will be by virtual call or by phone (your preference).

What will happen to the results of the trial?

At the end of the trial, any results will be analysed and published in a medical journal. Your personal details will remain strictly confidential. Research findings made available in any reports or publications will not include information that can directly identify you without your specific consent. An information sheet detailing the findings of the trial will be sent to your hospital medical team and they will be asked to share this with the participants of the trial.

What if I change my mind?

If you do decide to take part in VALUE, then you are still free to withdraw at any time without giving a reason. This will not affect your future care in any way. Your rights to access, change or move your information are limited, as we need to manage your information in specific ways in order for the research to be reliable and accurate. If you withdraw from the study, we will keep the information about you that we have already obtained. All data collected up to the point of withdrawal from the trial will be kept as part of the trial data.

If you have consented to be interviewed about your thoughts and experiences, you can change your mind at any point. If you change your mind after the interview is completed, the data collected from the interview will have been merged with information from other people's interviews and will not be removed.

How will we use information about you?

We will need to use information from you and from your medical records for this research project.

This information will include your initials, name, year of birth, demographics and contact details.

People will use this information to do the research or to check your records to make sure that the research is being done correctly.

People who do not need to know who you are will not be able to see your name or contact details. Your data will have a code number instead. All patients will be identified by a trial ID number which is added to the signed consent form. Those who consent to being contacted by the qualitative researcher will be identified by this unique number within the trial database.

Access to the database is restricted to those who work on the study only and requires a secure login.

Consent forms are kept within a restricted access folder for central monitoring before being destroyed. Those who consent to interview are included in a second restricted access folder for the qualitative reviewer to access and destroy.

We will keep all information about you safe and secure.

Once we have finished the study, we will keep some of the data so we can check the results. We will write reports in a way that no-one can work out that you took part in the study.

The Southampton Clinical Trials Unit will keep non-identifiable information about you for 15 years after the study has finished.

[Insert name of local NHS site] will keep identifiable information about you from this study for 15 years after the study has finished.

We will collect some demographic information from you to help establish similarities and patterns with other interviewees. This will be asked during the interview.

For the period of the study up to 3 years after its conclusion, your personal data (e.g., name, contact details and demographic data) will be stored at UoS, who are the acting "Data Processor" for this study, after which it will be destroyed according to the General Data Protection Regulations (GDPR). Some of your information might be temporarily stored on servers in the EU and USA and will be anonymised. They must follow data protection laws.

You can find out more about how we use your information at: https://www.southampton.ac.uk/ctu/about/index.page.

Optional interviews

What happens to the interview recording?

The interviewer will call and record the discussion using Microsoft Teams, which is a University of Southampton approved software. Microsoft Teams is compliant with a range of regulatory security standards.

The interview recording will be labelled with a code number not related to any of your personal information and securely sent through an online portal to McGowan Transcriptions who will type out everything discussed in the interview. The company used to do this has an agreement to keep everything said in the interview secret. Once typed out, we will remove any names, places etc which would identify you or any other individuals.

FUNDED BY
NIHR | National Institute for Health and Care Research

The digital recording and the typed-up record (transcript), identified only by the code number, will be kept in a secure folder within University of Southampton as per Research data management policy (https://www.southampton.ac.uk/~assets/doc/calendar/Research%20Data%20Management%20Policy.pdf).

Once transcripts have been error-checked, the original interview recording will be deleted.
We will analyse the text of the interview to identify the most important points. These will be compared with other interviews.

A scientific article will be written based on this information and will be sent to a scientific journal for publication. The results may also be presented in reports and conference presentations. Some selected quotes from your interview may be used in these publications, but your name and any identifiable information will never be mentioned. The transcripts will be kept for up to 10 years on a securely encrypted, password-protected university computer in case we need to review them.

If you wish to know more about the interviews, please email valueq@soton.ac.uk .

Consent Forms

A copy of your consent form, containing your name and initials, will be sent to the Southampton Clinical Trials Unit for confirmation of your consent. This form will be kept securely, as detailed above, for the duration of the trial.

If you agree to the interview, your contact details will be provided to Southampton CTU so that they can make arrangements with you directly.

What are your choices about how information is used?

You can stop being part of the study at any time, without giving a reason, but we will keep information about you that we already have.

We need to manage your records in specific ways for the research to be reliable. This means that we won't be able to let you see or change the data we hold about you.

If you agree to take part in this study, you will have the option to take part in future research using your data saved in this study.

Where can you find out more about how your information is used?

You can find out more about how we use your information:

- at www.hra.nhs.uk/information-about-patients/
- our leaflet available from https://www.southampton.ac.uk/ctu/about/index.page
- by asking one of the research team
- by sending an email to dataprotection@uhs.nhs.uk

Who is organising and funding the trial?

FUNDED BY NIHR | National Institute for Health and Care Research

The VALUE study is funded by the National Institute for Health and Care Research (NIHR) and sponsored by the University Hospital Southampton NHS Foundation Trust. The study has been ethically reviewed by the West of Scotland Research Ethics Committee.

Who do I contact if there is a problem?

If you have any questions, please contact your local study team:

[Named site nurse] Research nurse, [insert site name] on Tel [……]

[insert name site] PI, [insert PI name], Tel [insert number site specific]

If you are unhappy with the care, you received as part of the study you can contact:

Patient Advice and Liaison Service (PALS) on <site to add PALs number> or email <site to add PALS email>. It is very unlikely that anything will go wrong due to trial participation.

Thank you for taking the time to read this information sheet and to consider the study.

FUNDED BY NIHR | National Institute for Health and Care Research
