# Supplementary material for: Understanding the variation of modern endoscopic ultrasound use in patients with oesophageal cancer (VALUE): protocol for a multi-methods study
Source: BJR Open. 2025 May 21;7(1):tzaf012. doi: 10.1093/bjro/tzaf012 (PMC12145170; doi:10.1093/bjro/tzaf012)
Supplement: tzaf012_Supplementary_Data [file tzaf012_supplementary_data.zip › VALUE_Informed_Consent_Form_v2_24May2024_.pdf]

(To be printed on local headed paper)

INFORMED CONSENT FORM

Study Title: Understanding the variation of modern endoscopic ultrasound use in patients with oesophageal cancer: a multi-methods study

Patient Identification Number for this trial: ☐☐☐ — ☐☐☐☐☐☐☐

Name of Researcher:

	Please initial box
1. I confirm that I have read and understand the information sheet dated XX-XXX-2024 (version X) for the above study and I fully understand what is involved in taking part in this trial. I have had the opportunity to consider the information, ask questions and these have been answered satisfactorily.	INITIAL
2. I understand that my participation is voluntary and that I am free to withdraw at any time without giving any reason and without my medical care or legal rights being affected.	INITIAL
3. I understand that should I withdraw from the study then the information collected about me up to this point may still be used for the purposes of achieving the objectives of the study.	INITIAL
4. I consent to the storage of personal information (including electronic) for the purposes of this study. I understand that any information that could identify me will be kept strictly confidential and that no personal information will be included in the study report or other publication.	INITIAL
5. I give permission for a copy of my consent form to be sent to the Southampton Clinical Trials Unit, where it will be stored securely, to allow confirmation of my consent.	INITIAL
6. I understand that relevant sections of my medical records, and data collected during the study, may be looked at by individuals from the Sponsor or their delegates, or from the NHS Trust where it is relevant to my taking part in this research. I give permission for these individuals to have access to my records.	INITIAL
7. I understand that I shall not benefit financially even if future research leads to the development of new treatments or medical tests.	INITIAL

FUNDED BY NIHR | National Institute for Health and Care Research

		Yes	No
8.	**OPTIONAL:** I agree to my anonymised data being used in future ethically approved research (in the UK and abroad), which may be shared anonymously with other researchers.	INITIAL	INITIAL

9.	**OPTIONAL:** I agree to be contacted by a researcher from the Southampton Clinical Trials Unit (SCTU) to discuss my experience of EUS.	INITIAL	INITIAL

Yes, to schedule a time to discuss my experience please contact me by (tick as appropriate):

Phone ☐ Phone number:

Most convenient day(s) and time(s) to call:

OR

Email ☐ Email address:

10. **I agree to take part in the VALUE study.**	INITIAL

Name of Patient	Signature	Date
Name of researcher taking consent	Signature	Date

When completed:

- 1 for participant;
- 1 to be kept in medical notes;
- 1 (original) for investigator site file;
- One copy to be emailed to monitorSCTU@soton.ac.uk using safesend or encrypted mail at monitorSCTU@uhs.nhs.uk to allow for central monitoring

FUNDED BY NIHR | National Institute for Health and Care Research
